# Weight restoration on a high carbohydrate refeeding diet promotes rapid weight regain and hepatic lipid accumulation in female anorexic rats

**DOI:** 10.1186/s12986-016-0077-y

**Published:** 2016-03-01

**Authors:** Erin D. Giles, Jennifer Hagman, Zhaoxing Pan, Paul S. MacLean, Janine A. Higgins

**Affiliations:** Center for Human Nutrition, University of Colorado Anschutz Medical Campus, Aurora, CO USA; Division of Endocrinology, Diabetes and Metabolism, School of Medicine, University of Colorado Anschutz Medical Campus, Aurora, CO USA; Department of Psychiatry, School of Medicine, University of Colorado Anschutz Medical Campus, Aurora, CO USA; Biostatistics Core, Children’s Hospital Colorado Research Institute, Aurora, CO USA; Department of Pediatrics, School of Medicine, University of Colorado Anschutz Medical Campus, Aurora, CO USA

**Keywords:** Anorexia nervosa, Refeeding, Indirect calorimetry, Energy expenditure, Activity-based anorexia, Carbohydrate, Diet, Rat, Clinical

## Abstract

**Background:**

There is currently no standard clinical refeeding diet for the treatment of anorexia nervosa (AN). To provide the most efficacious AN clinical care, it is necessary to define the metabolic effects of current refeeding diets.

**Methods:**

An activity-based model of anorexia nervosa (AN) was used in female rats. AN was induced over 7d by timed access to low fat (LF) diet with free access to a running wheel. Plasma hormones/metabolites and body composition were assessed at baseline, AN diagnosis (day 0), and following 28d of refeeding on LF diet. Energy balance and expenditure were measured via continuous indirect calorimetry on days −3 to +3.

**Results:**

AN induction caused stress as indicated by higher levels of corticosterone versus controls (*p* < 0.0001). The rate of weight gain during refeeding was higher in AN rats than controls (*p* = 0.0188), despite lower overall energy intake (*p* < 0.0001). This was possible due to lower total energy expenditure (TEE) at the time of AN diagnosis which remained significantly lower during the entire refeeding period, driven by markedly lower resting energy expenditure (REE). AN rats exhibited lower lipid accumulation in visceral adipose tissues (VAT) but much higher liver accumulation (62 % higher in AN than control; *p* < 0.05) while maintaining the same total body weight as controls. It is possible that liver lipid accumulation was caused by overfeeding of carbohydrate suggesting that a lower carbohydrate, higher fat diet may be beneficial during AN treatment. To test whether such a diet would be accepted clinically, we conducted a study in adolescent female AN patients which showed equivalent palatability and acceptability for LF and moderate fat diets. In addition, this diet was feasible to provide clinically during inpatient treatment in this population.

**Conclusion:**

Refeeding a LF diet to restore body weight in female AN rats caused depressed TEE and REE which facilitated rapid regain. However, this weight gain was metabolically unhealthy as it resulted in elevated lipid accumulation in the liver. It is necessary to investigate the use of other diets, such as lower carbohydrate, moderate fat diets, in pre-clinical models to develop the optimal clinical refeeding diets for AN.

## Background

Anorexia nervosa (AN) is a psychiatric illness characterized by body dissatisfaction accompanied by restriction of energy intake leading to a significantly low body weight, and persistent behaviors, including high levels of physical activity, that interfere with weight gain [1]. Individuals with AN become severely malnourished and develop significant medical complications [2, 3]. Although weight restoration is a primary intervention for AN, along with psychotherapy and behavioral interventions, there has been little research on optimal refeeding strategies. There is currently no standard, evidence-based clinical refeeding diet for the treatment of anorexia nervosa (AN) and nutritional treatment is focused primarily on calories and less on diet composition [4]. However, studies in both rats and humans show that diet composition can affect the rate of weight regain following weight loss, impact the ability to maintain at a target weight, and dramatically alter metabolic health [5–7]. Therefore, in order to provide the most efficacious clinical care for AN patients, it is necessary to define the effect of current refeeding diets on markers of metabolic health and use this information to develop optimal AN refeeding diets. Our ultimate goal is to achieve metabolically healthy weight gain in this population.

Given the low overall prevalence of AN in the population and the challenges in treating the illness, it would be difficult to test multiple different refeeding diets in AN patients. However, there is an established rodent model of AN that can be used for this purpose. Activity-based anorexia (ABA) is a well-established paradigm in which restricted time with access to food combined with free access to exercise causes hyperactivity, reduced energy intake, and, ultimately, anorexia in rats [8] (reviewed in [9, 10]) and to a lesser extent mice (reviewed in [11]). Most early studies of the ABA model utilized male rats, provided access to food for 1–2 h per day, and defined anorexia as a body weight at 65–75 % of free feeding or initial body weight. However, because AN in humans is most common in females, there has been a shift towards the use of female rats in the ABA model, primarily to study brain structure and neuronal biology/signaling in AN [12–15]. We endeavored to use recently published data about the diet and physical activity characteristics of female adolescent AN patients [16] to refine the ABA model in pubertal female rats to more closely mimic the human condition.

The goals of this study were to: 1) define the metabolic effects of a clinical refeeding diet on markers of metabolic health in a modified rodent model of AN and, 2) to develop a rat model of AN that closely mimics the human condition which could be used to screen more effective refeeding diets in the future. We hypothesized that, during the refeeding phase, AN rats would exhibit “catch-up growth” with preferential deposition of ingested energy into adipose depots [17–19], especially visceral adipose, indicative of a higher metabolic risk for cardiovascular disease and associated co-morbidities.

## Methods

### Rat energy balance study

#### Animals and housing

Female Sprague–Dawley rats (104-140 g; 5 weeks of age) possessing a polygenic predisposition for leanness and increased physical activity (lean/activity-prone, LAP) were purchased from Taconic Farms Inc. (Hudson, New York). Rats were housed individually in suspended wire cages at the University of Colorado Anschutz Medical Campus. All rats had free access to water in a temperature (22–25 °C) and humidity (30–40 %) controlled room with a 12 h light–dark cycle (lights off at 1300). All procedures were approved by the institutional animal care and use committee. Rats were allowed to acclimatize for 5 days, during which they had free access to a moderate fat (MF) diet (25 % kcal fat, 21 % kcal protein, 54 % kcal carbohydrate; Research Diets #D07091301, New Brunswick, NJ).

### Activity-based anorexia model

#### Anorexia nervosa induction

Following acclimatization, rats were randomly assigned to either the anorexia nervosa (AN) or control groups. AN rats were moved to wire mesh cages (13 x 25 x 13 cm) attached to a running wheel of 1.1 m circumference, and were given free access to the wheel for 23 h per day. The door separating the cage and the running wheel was closed for 1 h each day (1400–1500 h), during which time animals were given *ad libitum* access to a low fat (LF) diet (12 % kcal fat, 20 % kcal protein, 68 % kcal carbohydrate; Research Diets #D11724, New Brunswick, NJ). Control rats had the same *ad libitum* access to the LF diet for 1 h per day, but remained in suspended wire cages without access to a running wheel for the entire study. Food intake and body weight were measured daily.

#### Anorexia

Rats were identified as anorexic (day 0) when their body weight decreased to ≤75 % of initial weight. 75 % of initial body weight was chosen as the threshold for AN as we previously found that this was the mean percent ideal body weight of female adolescent AN patients who presented for inpatient medical care at Children’s Hospital Colorado [16]. For control animals, day 0 was defined as the seventh day of timed feeding.

#### Weight regain

Access to running wheels was blocked and refeeding began on day 1 in all animals. All rats were refed on LF diet (described above). This diet is similar in composition to the standard refeeding diet used in the treatment AN patients at the Children’s Hospital Colorado Eating Disorders Program [16].

For the AN group, refeeding for the first 3 days was based on each animal’s energy intake measured on day 0. On days 1 and 2, AN rats were given 200 % of their energy intake at day 0. This was increased to 300 % on day 3, followed by *ad libitum* feeding for the remainder of the study. This staged re-feeding protocol for the AN group was conducted to mimic the clinical dietary paradigm currently used in adolescent AN patients. In the clinical program, caloric intake is increased by 250 kcal/d until patients reach a weight gain of 1 kg/wk. This paradigm was accelerated in the rat model by increasing caloric intake more quickly for two reasons: 1) at AN diagnosis, rats were frail so we wanted provide an opportunity for rapid recovery, and 2) to measure the effect of this approach on energy balance while rats were undergoing indirect calorimetry.

#### Indirect calorimetry

A metabolic monitoring system (Columbus Instruments, Columbus, OH) was used to assess energy intake and total energy expenditure in a subset of rats from the third day of timed feeding (anorexia induction; d-3) through three days of refeeding (d + 1, +2, and +3). Rats were randomly chosen for calorimetry, as the metabolic monitoring system did not have space to accommodate all rats used in this study. The multi-chamber indirect calorimetry system allows for the continuous monitoring of up to eight rats, obtaining measurements of oxygen consumption (vO_2_) and carbon dioxide production (vCO_2_) from each chamber every 16 min [19–21]. The chambers also allow for the collection of daily urine, feces, and food spillage. Daily total energy expenditure (TEE) was calculated from gas exchange measurements acquired over the 24-h period using the Weir equation (MR = 3.941 **•** vO_2_ + 1.106 **•** vCO_2_ – 2.17 **•** N, where N represents urinary nitrogen) [22]. All data is expressed as kcal/day.

#### Body composition analysis

Body composition was determined by quantitative magnetic resonance (qMR; Echo MRI Whole Body Composition Analyzer; Echo Medical Systems, Houston, TX). Measurements were obtained on the first day of the timed feeding protocol, day 0, day 7, and at the end of study (day 30). Liver qMR measurements were taken at end of study, and only livers with weights ≥5.5 g were used for analysis due to the limits of sensitivity of our instrumentation.

#### Plasma and urine analyses

Urine samples were taken from the experimental and control group rats every 48 h while they were in the metabolic monitoring system. Urine was assayed for corticosterone, creatinine, and urea using a commercially available ELISAs (Fisher Scientific, Lafayette, CO). Urinary nitrogen was estimated from measurements of urea and creatinine, as previously described [20, 23].

Tail vein blood was collected under isoflurane anesthesia (MWI Veterinary Supply, Meridian, ID) from the animals at baseline, day 0, and end of study (day 28). Blood draws were taken after the animals had been fasting for 16–19 h (2.5 to 5.5 h prior to the next feeding period). The plasma was assayed using commercially available ELISAs for insulin (Alpco, Salem, NH), glucose (Fisher Scientific, Lafayette, CO), and leptin (Millipore Corp. Billerica, MA).

### Clinical feasibility and proof of concept study

To determine the feasibility of feeding a diet higher in fat to patients with AN, we conducted 1) a diet palatability and acceptance study, and 2) a proof of concept study. These studies were conducted according to Declaration of Helsinki guidelines and all procedures were approved by the University of Colorado Anschutz Medical Campus Combined Institutional Review Board (COMIRB). All participants provided written assent with the written informed consent of a parent or guardian. Female subjects 12–21 years of age with a diagnosis of AN (restricting or binge/purge type) were recruited. All subjects were required to be undergoing inpatient eating disorder treatment to ensure that the most severely affected individuals are capable of eating a modified diet. All subjects had to be able to read and comprehend English at a 4th grade level.

- Diet Palatability and Acceptance in AN patients
  Eight female inpatients, aged 12 to 21 years, diagnosed with AN were asked to rate the taste and acceptability of two different diets presented in random order: 1) the current clinical AN refeeding diet based on a lower fat, moderate protein diet (15 % protein, 26 % fat, 59 % carbohydrate), or 2) a moderate fat, high protein test diet (20 % protein, 40 % fat, 40 % carbohydrate). The single, acute meal tests replaced the participant’s normal lunch. The macronutrient content of the clinical diet was calculated based on analysis (Nutrition Data System for Research; NDSR, University of Minnesota, Nutrition Coordinating Center) of photographs of all breakfasts, lunches, and daytime snacks served on the EDU over two consecutive days. Subjects had free access to water during the meals. All portions were weighed before and after consumption to measure the amount of food eaten.
  The main endpoint was taste assessment, based on a validated five face scale [24]. Participants were asked to complete a Likert scale for 1) taste, and 2) “would you eat this again?” after consuming both the LF and MF diets. Scores were analyzed in two ways: the raw distribution of the five faces, and a two-group score (very good + good + average representing satisfactory taste and bad + very bad, representing unsatisfactory taste). Percent distribution of taste satisfaction score was calculated with 95 % confidence interval estimates.
- Proof of Concept Case Study
  To test whether the MF diet (20 % protein, 40 % fat, 40 % carbohydrate) could logistically be provided by the food services department at the hospital, and that it could be consumed consistently on an inpatient basis, we also performed a proof of concept study in which one patient followed the test MF diet for 10 consecutive days. The MF diet utilized only foods available from the hospital food service department.
  The participant was a 13 year old female with a 9 month documented duration of AN. She was at 72 % of ideal body weight at the commencement of the study (BMI 12.4). The participant, working with a research nutritionist, self-selected daily menus from the complete option list according to the nutrition intervention utilized in the treatment program. The menu for this subject was altered to provide over 120 menu options that would fulfill the dietary requirements of the study. All menus met clinical care targets for daily caloric goal. All other aspects of standard psychiatric and medical care were provided during this study.

### Statistical analysis

SAS 9.3 was used for all the analyses. Repeated measures ANOVA by linear mixed effects model was used to make with-group and between-group comparisons for energy expenditure, energy balance, hormone, metabolite and body composition data collected prior to and at diagnosis and during re-feeding phases. A quadratic linear mixed effects model with random intercept, linear and quadratic coefficients was used to model daily body weight of rats during the refeeding phase. Two sample *t*-test was used to compare two group for data at rat sacrifice. No adjustment for multiple comparisons was applied. A *p*-value less than 0.05 was deemed to be statistically significant.

## Results

### Rat energy balance study

#### EI, wheel use, and body weight

**Fig. 1:**
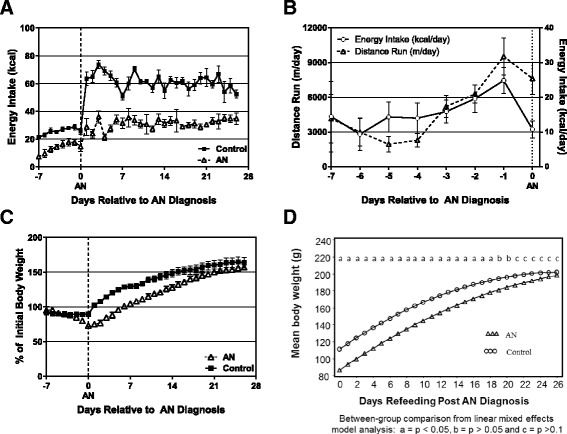
Energy intake, running activity, and body weights for AN and control rats. **a** Energy Intake (EI) in control and AN rats for the duration of the study; **b** Distance run (left axis; m/d) and energy intake (right axis; kcal/d) during development of AN; **c** Body weights (expressed as % of initial body weight) for AN and control rats. **d** Body weights for AN and control rats with the results of mixed model analysis. For A-C, data shows group mean ± SEM

Energy intake (EI; kcal/d) in the AN group decreased by approximately 50 % from day −2 to diagnosis of AN (day 0), and AN rats were consuming significantly fewer calories on day 0 than controls (*p* = 0.0002; Fig. 1a). Wheel running increased significantly in the 4 days leading up to AN diagnosis (Fig. 1b). This combination of increased physical activity and decreased energy intake precipitated the decline to an anorexic body weight, defined as 75 % of initial weight (90.1 ± 2.8 g), in 4 to 7 days (Fig. 1c). 75 % of initial body weight was chosen as the threshold for AN because we previously found that this was the mean percent ideal body weight of female adolescent AN patients who presented for inpatient medical care [16]. When given the same timed access to food without running wheels, control animals remained weight stable over the 7 day lead-in period (Fig. 1c).

In the AN group, refeeding was modeled after the staged refeeding protocol currently used in adolescent AN patients at our hospital. Thus, on days 1 and 2 of refeeding, rats were provided 200 % of their intake on d0. This was increased to 300 % on d3, and ad libitum intake for the remainder of the study. Conversely, control rats were allowed ad libitum food access for the entire refeeding period. In a mixed model analysis, a model consisting of group indicator, linear, quadratic terms and linear by group interaction as fixed effects best described the weight regain (Fig. 1d). Both AN and control rats gained a significant amount of weight over the weight re-gain period, but the rate of weight gain was significantly greater in the AN rats than controls (*p* = 0.0188). By d19 of refeeding, the body weights of the AN rats (180.9 ± 6.0 g) were no longer statistically different from controls (194.3 ± 5.7 g; Fig. 1c).

#### Energy expenditure and energy balance

**Fig. 2:**
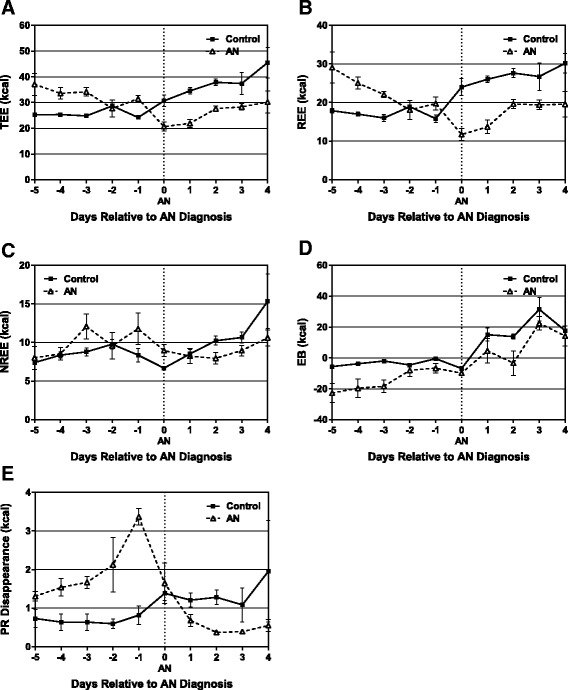
Energy expenditure, energy balance, and protein disappearance during the development of AN and the early refeeding period. **a** Total Energy Expenditure (TEE), which was also divided into its **b** Non-resting (NREE) and **c** Resting (REE) components. **d** Energy Balance (EB) **e** Protein Disappearance. All data shows group mean ± SEM

Total energy expenditure (TEE) gradually declined in the 5 days leading to the AN diagnosis (Fig. 2a), a result of a declining resting energy expenditure (REE, Fig. 2b). During this time, nonresting energy expenditure (NREE) increased (Fig. 2c) as physical activity increased (Fig. 1b), until energy intake precipitously declined (Fig. 1a). At the time of AN diagnosis, REE reached a distinct nadir, while NREE remained relatively high. AN rats were in a negative energy imbalance during the entire lead-in period. The adaptive response to decrease REE minimized the energy imbalance in AN rats to −10 kcal at the time of diagnosis. In addition, protein disappearance increased during the days leading up to AN diagnosis (Fig. 2e), implying that these animals are breaking down lean body mass in attempt to maintain body weight. In contrast, the control group were in relative energy balance throughout the entire lead-in period (Fig. 2d) with little variation in TEE, REE, and NREE, consistent with stable body weight (Fig. 1c).

Over the first 4 days of refeeding, TEE increased in both the AN and control groups, but TEE was lower in AN rats compared to controls (Fig. 2a). This decrease in TEE is explained primarily by the lower REE in the AN group (Fig. 2b). NREE did not change significantly in either the AN or control group during the first few days of refeeding, despite the fact that wheel running activity was stopped. This suggests that the increased food intake during this time, and the resulting increase in thermic effect of food (TEF), offset the decrease in activity thermogenesis, resulting in no change in NREE (Fig. 2c). As a result, EB increased in both the AN and control group during the refeeding period, with no difference between the groups (Fig. 2d).

EI was lower in AN than controls rats (EI = 33, 35, and 71 % of control on days 1, 2, and 3 of refeeding, respectively; *p* < 0.0001) resulting in lower thermic effect of food in the AN group during this time. Despite this lower EI in AN rats, TEE was so much lower that energy balance was the same for the AN and control groups on days 2 and 3 of refeeding (Fig. 2d). The differences in TEE between AN and control rats during refeeding was primarily due to suppression of REE (Fig. 2b) with a much lower contribution from NREE (Fig. 2c).

#### Hormone and metabolite responses

**Fig. 3:**
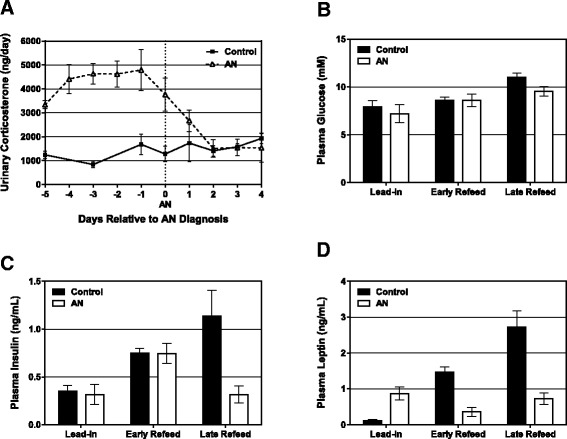
Metabolite and hormone measurements. **a** Urinary corticosterone, **b** plasma glucose, **c** plasma insulin, and **d** plasma leptin concentrations during the development of AN and the refeeding period. All data shows group mean ± SEM

The ABA protocol induced a significant stress response, as demonstrated by significantly higher levels of urinary corticosterone in AN rats, compared to controls (*P* < 0.0001; Fig. 3a). Corticosterone levels began to decline immediately with the cessation of exercise and increased food intake, and were not significantly different than controls by d1 of refeeding (Fig. 3a).

Plasma glucose, insulin and leptin concentrations were measured in the fasted state at three time points: 1) at baseline, prior to the start of the AN protocol; 2) during the early refeeding period (d2 to 14 of refeeding); and 3) at end of the study. Blood draws were not performed at AN diagnosis (day 0) as the animals were too fragile. Glucose levels increased over time (Fig. 3b; *p* < 0.001), as was expected given that all rats were overfeeding and in a positive energy imbalance, but there were no differences between groups. Plasma insulin levels also increased over time in the control, but not AN rats, and, at the end of study, insulin levels were significantly lower in the AN group when compared to controls (Fig. 3c; *p* < 0.05).

**Fig. 4:**
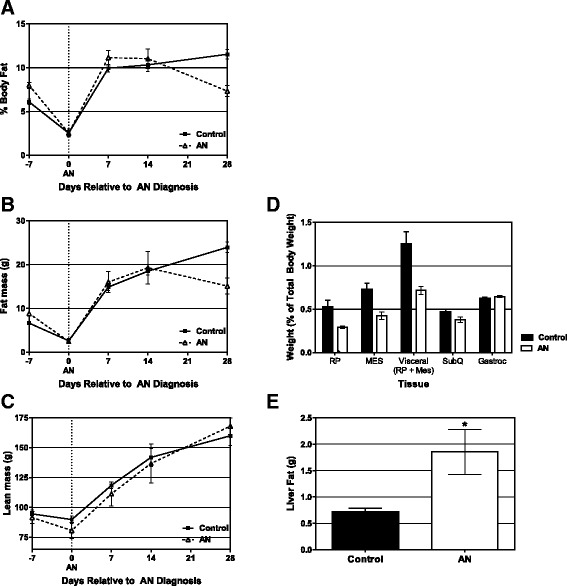
Body composition and regional adiposity of AN and control rats. **a** Percent body fat, **b** Absolute fat mass (g), and **c** Absolute lean mass were measured by qMR for AN and control rats at weekly intervals throughout the study. At the end of the study, **d** Regional fat distribution was assessed by weighing each fat pad and expressing the weight as a % of totally body weight. **e** Fat (g) in the liver was measured by qMR at end of study. All data shows group mean ± SEM

As expected, leptin levels increased in the control animals (Fig. 3d) as body fat increased (Fig. 4a). AN rats, however, had lower leptin levels in the early refeeding phase, despite having the same body weight and fat mass as control rats. At the end of the study, leptin was 3.7 fold lower in the AN rats compared to control rats, which is lower than what would be expected based on the 1.6 fold lower body fat.

#### Body composition

At the time of AN diagnosis (day 0) there was no difference in body fat or lean body mass between the two groups (Fig. 4a-b) despite a significantly lower body mass in the AN animals. Thus, while both access to a running wheel and timed food access are required for the development of AN, timed food access alone (control rats) led to a decrease in body fat without a significant change in total body weight. During the weight regain period, body fat increased significantly in both the AN and control groups (Fig. 4a-b). However, fat gain plateaued in the AN group during the second week of refeeding, but continued to increase in the control animals. As a result, AN rats gained significantly less fat over the follow-up period (day 0 vs 28, *p* < 0.001), perhaps an indication that they are “defending” their lower body weight. At the time of sacrifice, the amount of subcutaneous fat was not different between control and AN rats but visceral fat was significantly lower in the AN rats relative to controls (*p* = 0.02, Fig. 4d), suggesting that a decrease in visceral fat was primarily responsible for the lower total body fat in the AN group. Refeeding increased liver fat by approximately 40 % in AN rats relative to controls (0.7 vs 1.9 g in AN and controls, respectively; *p* < 0.05, Fig. 4e).

### Clinical feasibility and proof of concept study

#### Clinical acceptance of a diet higher in fat and protein

The average macronutrient content of the meal plan (selected by parents from a specialized menu, with dietician support and oversight) in the clinical program for adolescents with AN was 15 % protein, 26 % fat, and 59 % carbohydrate. Meals accounted for 64 % of total calories (16 % protein, 31 % fat, and 53 % carbohydrate), while snacks accounted for the remaining 36 % of total calories (11 % protein, 21 % fat, and 68 % carbohydrate).

**Fig. 5:**
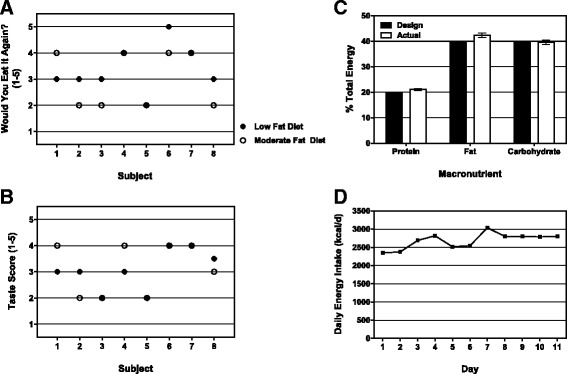
Lower carbohydrate, higher fat diet acceptability and feasibility in female adolescent AN patients. Eight female inpatients diagnosed with AN rated the taste and acceptability of the standard LF diet, and a test diet high in protein with moderate fat (MF) using a validated five face scale. **a** Results of the face scale for willingness to consume the diet in the future and **b** taste after consuming both the LF and MF diets. The MF diet utilized only foods available from the hospital food service department. Daily menus from the complete option list for MF diet were analyzed for **c** macronutrient targets ((20 % protein, 40 % fat, 40 % carbohydrate) and **d** fidelity to daily caloric goals

To follow up on the finding of increased liver fat observed during the rat refeeding model, and to explore the possibility of feeding a different “test diet” to patients with AN, which might decrease the risk of accumulation of liver fat during refeeding, we conducted feasibility studies utilizing a moderate fat (MF) diet. The current LF (15 % protein, 26 % fat, 59 % carbohydrate) and test MF (20 % protein, 40 % fat, 40 % carbohydrate) diets were ranked the same for both taste and palatability (Fig. 5a and b). There was no difference in the energy consumption between the two meals as participants consumed all food presented to them under both conditions. These data suggest that feeding a MF diet would be possible in this population. In addition, a single inpatient admitted to the clinical program was fed a dietician-directed, self-selected MF diet for 10 consecutive days proving that it is indeed possible to plan meals with a higher fat, higher protein and lower carbohydrates clinically in adolescents with AN during the refeeding period (Fig. 5c-d).

## Discussion

The primary goal of this study was to develop a rat model that can be used to evaluate refeeding strategies and potentially improve the process of weight restoration during the treatment of AN. Specifically, it is likely that different dietary regimens could be used to strengthen the biological response to refeeding and potentially improve weight regain and maintenance in this population. Thus, developing a model that mirrors the human condition was of critical importance. Our model, which combined a classic exercise induced anorexia paradigm (free access to a running wheel with timed access to food; [12]) with the addition of using female adolescent rats genetically predisposed to leanness, led to the development of AN within 4–7 days and was characterized by a loss in both total body mass and body fat. Unlike data from Dixon et al. [12], rats in the AN group in this study ate significantly less food during the AN induction phase than the control group. In addition, increased physical activity preceded any decrease in energy intake by several days which mirrors the development of human AN in which increased physical activity may be the first apparent symptom prior to onset of dramatic weight loss [25].

When AN rats were compared to control animals, refeeding on a standard LF diet caused comparable increases in total body weight and subcutaneous fat accumulation. However, leptin concentrations were lower in the AN rats compared to controls and were also lower in AN rats than would be expected based on total body fat. This phenomenon of leptin concentration underestimating body fat stores is similar to that seen in rodent models of obesity whereby formerly obese rats that lose weight do not have restored leptin concentrations after weight regain [23]. In our AN model, however, the effects are more dramatic than in the models of obesity. Although the underlying mechanism for this effect is unknown, the fact that the same amount of adipose tissue is secreting different amounts of leptin suggests that the adipo-insulin and adipo-leptin axes are perturbed in these animals.

Despite similar increases in total body fat, AN rats exhibited dramatically higher liver fat compared to controls. It is likely that the high accumulation of liver fat is due, at least in part, to increased de novo lipogenesis from excess carbohydrate intake during the refeeding period [26]. In addition, fat gain appeared to plateau in the AN rats during the second week of refeeding. In models of obesity-associated weight loss and weight regain, exercise appears to decrease the formation of new adipocytes via adipogenesis [27–29]. Thus, we would speculate that the exercise during the AN development phase, combined with the increase in caloric intake during the refeeding period, resulted in decreased formation of new adipocytes early in the regain period. If this is the case: 1) existing adipocytes likely reached their storage capacity during the first week of refeeding, and, after this time, excess nutrients were deposited in non-adipose storage sites, such as the liver, and 2) the lower number of total adipocytes may, in part, explain the lower leptin concentrations observed in AN rats. An alternative explanation is that the cessation of exercise could also contribute to hepatic de novo lipogenesis and the noticeable increase in lipid accumulation in the liver of AN rats, as cessation of daily exercise has been shown to promote hepatic steatosis in hyperphagic/obese rats [30]. Future studies could include use of tracers to directly address the etiology of lipid accumulation. Regardless of the underlying mechanism, this increase in fat accumulation in the liver is cause for concern, as liver fat is associated with insulin resistance [31] and the development of type 2 diabetes, independent of total adiposity [32].

It is not known if patients with AN develop increased liver fat during the refeeding process. Further research is needed to determine if human subjects undergoing weight restoration have similar findings. To counter the negative effects of a LF diet on fat accumulation in the liver, observed on the AN group, it may be optimal to adjust the composition of the diet during weight restoration in AN to a diet with lower carbohydrate and increased fat content. Although previous AN research has shown that the percentage of fat and protein is not lower in acute inpatient AN than in age and sex matched adolescents, the absolute amounts of fat and protein intake are low [16]. Thus, it is possible that increasing the fat and protein content of an AN refeeding diet may be beneficial. Indeed, during the timed food restriction, induction phase of ABA, a high fat diet can prevent AN in rats [33, 34]. This effect is due to both the preservation of caloric intake during the AN induction phase and decreased physical activity relative to standard rat chow which is a high carbohydrate food [34].

We would hypothesize that a diet higher in both fat and protein, and lower in carbohydrate, could ameliorate the fat accumulation observed in the liver in the current model and could also increase the rate of weight gain (by decreasing de novo lipogenesis with a concomitant decrease in TEE). This strategy would agree with the recommendation to limit AN refeeding diets to less than 40 % of calories from carbohydrate to reduce the risk of refeeding syndrome [35]. However, if both fat and protein are to be used to replace dietary carbohydrate, caution must be exercised as there are selected reports of hyperammonemia with high protein intake after substantial weight loss [36]. Although these data are from case reports and are rare in AN patients [37], it is important that refeeding diets with higher protein contents be tested in a pre-clinical model, such as that described herein, before being administered clinically.

Although it is possible that higher fat and protein refeeding diets may be beneficial, it is commonly believed that AN patients have an aversion to fat although the data supporting this assumption are equivocal [38]. To address this possible concern, we conducted a feasibility study in adolescent females with AN, which showed that a MF diet is both acceptable and palatable to patients with AN and that such a diet can be delivered successfully in an inpatient clinical setting. In the single patient with AN who consumed the MF diet for 10 consecutive days during weight restoration, all study parameters for macronutrient content were achieved in addition to following the program’s standard of care guidelines for calorie changes over time, rate of weight gain, monitoring food avoidance behaviors and enhancing reward systems, and behavioral motivation, etc.

The measured average macronutrient content of the clinical refeeding diet utilized in the treatment program in this study matches that for a similar sample of subjects measured retrospectively at the time of medical hospitalization for malnutrition and bradycardia [16]. As part of treatment, parents work closely with dieticians to plan the patient’s meals based on a specific number of protein, carbohydrate, and satiety (fat) foods to achieve the daily caloric goal for weight restoration so the macronutrient content of these inpatient diets may be somewhat limited by this process.

The onset of AN and maintenance of the illness is associated with decreased caloric intake over a sustained period of time, ranging from months to years. Further studies are needed to determine optimal macronutrient composition and rate of weight gain for the weight restoration phase of treatment of AN, and if sustained change in dietary composition improves outcomes. It is possible that the cognitive changes associated with AN (body image distortion, fear of fat, harm avoidance and cognitive rigidity) may also respond to changes in dietary composition during weight restoration.

## Conclusion

We have made subtle changes to the standard rat ABA model such that the development of AN more closely mimics the human condition. In this model, refeeding a LF diet to restore body weight in AN rats caused depressed TEE and REE which facilitated rapid regain. However, this weight gain was metabolically unhealthy as it caused elevated lipid accumulation in the liver. Future studies should explore if this finding also occurs in human subjects with AN undergoing weight restoration. Further, it is necessary to investigate the use of other diets with different macronutrient ratios, such as lower carbohydrate, higher fat diets, in pre-clinical models to develop the optimal clinical refeeding diets for the weight restoration phase of AN treatment. Our preliminary data suggest that such diets are acceptable and feasible to deliver clinically in an inpatient hospital setting.

## Ethics approval and consent to participate

Rodent studies were approved by the University of Colorado Anschutz Medical Campus Institutional Animal Care and Use Committee (IACUC). Human studies were conducted according to Declaration of Helsinki guidelines and all procedures were approved by the University of Colorado Anschutz Medical Campus Combined Institutional Review Board (COMIRB). All participants provided written assent with the written informed consent of a parent or guardian.

## Consent for publication

Consent to publish has been obtained from all individuals or their parent or legal guardian.

## Abbreviations

AN: anorexia nervosa
ABA: activity based anorexia
BMI: Body Mass Index
EDU: Children’s Hospital Colorado Eating Disorders Unit
LAP: lean/activity-prone
LF: Low fat diet (12 % kcal fat)
MF: Moderate fat diet (25 % kcal fat)
MR: Metabolic rate
N: Urinary nitrogen
NDSR: Nutrition Data System for Research
NREE: non-resting energy expenditure
qMR: Quantitative Magnetic Resonance
TEE: total energy expenditure
vCO_2_: carbon dioxide production
vO_2_: oxygen consumption
